# Case Report: Metastatic breast cancer to the gallbladder

**DOI:** 10.12688/f1000research.23469.1

**Published:** 2020-05-11

**Authors:** Giulia Missori, Francesco Serra, Giorgia Prestigiacomo, Andrea Aurelio Ricciardolo, Lucio Brugioni, Roberta Gelmini

**Affiliations:** 1 Department of General Surgery, University of Modena and Reggio Emilia, Modena, Italy, 41124, Italy; 2 Department of Emergency Medicine, University Hospital Modena, Modena, Italy, 41124, Italy

**Keywords:** Emergency surgery, Breast cancer, Cholecystitis

## Abstract

Cholecystitis is one of the leading causes of emergency surgical interventions; the occurrence of metastases to the gallbladder is rare and has only been reported in the literature exceptionally. Metastatic breast cancer to the gallbladder is even less frequent; in fact, breast cancer usually metastasizes to bone, lung, lymph nodes, liver and brain. We report the case of an 83-year-old female patient with a previous history of breast surgery with axillary dissection in 1997, followed by adjuvant chemotherapy due to invasive ductal carcinoma of the left breast. The patient was admitted at the emergency department for sepsis and an episode of acute kidney failure, anuria and fever. Right-upper quadrant abdominal pain triggered by food intake and abdominal tenderness was also present, placing the diagnostic suspicion of biliary sepsis due to acute cholecystitis. The histological examination of the surgical specimen highlighted the presence of metastasis from an infiltrating ductal breast carcinoma with positive hormone receptors. We also report here the results of a review of the literature looking at articles describing cases of gallbladder metastasis from breast cancer.

## Introduction

Cholecystitis is one of the leading causes of emergency surgical interventions. The diagnosis of acute cholecystitis is usually based on physical examination, laboratory tests and abdominal ultrasound. The surgical options for cholecystitis are either open and laparoscopic cholecystectomy; the latter is nowadays considered the gold standard of treatment. Surgical specimens must be sent for histopathological examination to rule out cancer ^1^.

The occurrence of metastases to the gallbladder is rare and has only been reported in the literature exceptionally ^2^. Primary tumors can metastasize to the gallbladder either by proximity, such as hepatocellular carcinoma and pancreatic carcinoma, or by blood diffusion ^3^.

Chan reported, in a series of 7910 cholecystectomy specimens, that 36 cases of metastatic carcinoma were found, more often secondary to the stomach, lower gastrointestinal tract, liver, kidney or skin (malignant melanoma) cancer ^4^. Another more recent study shows that metastasis to the gallbladder accounted for 7/225 (3.1%) of the incidental gallbladder malignancies ^5^. Metastasis from breast cancer to the gallbladder is even less common; in fact, breast cancer usually metastasizes to bone, lung, lymph nodes, liver and brain.

We describe here the case of a patient who underwent cholecystectomy for acute cholecystitis with gallbladder metastasis from breast cancer. Subsequently, we present the results of a literature search concerning this disease.

## Case report

We report the case of an 83-year-old female patient with a previous history of breast surgery with axillary dissection in 1997, followed by adjuvant chemotherapy due to invasive ductal carcinoma of the left breast. The family history was negative for neoplastic diseases, both mammary and belonging to the gastrointestinal tract. Oncological follow-up was negative, and the patient considered disease-free for almost 15 years. During 2012, an X-ray of the spine, performed for the appearance of lumbar pain, revealed the presence of vertebral metastases. The patient was treated with radiotherapy and spinal stabilization. In addition to this, a deep venous thrombosis episode was reported in 2017, and treated with anticoagulant therapy. In the same year, multiple myeloma associated with mild chronic kidney disease was diagnosed. Neither myeloma nor kidney disease had requested specific treatments.

In July 2018, the patient was admitted to the emergency department for sepsis and an episode of acute kidney failure, anuria and fever. Right-upper quadrant abdominal pain triggered by food intake and abdominal tenderness was also present, placing the diagnostic suspicion of biliary sepsis due to acute cholecystitis.

**Figure 1.**
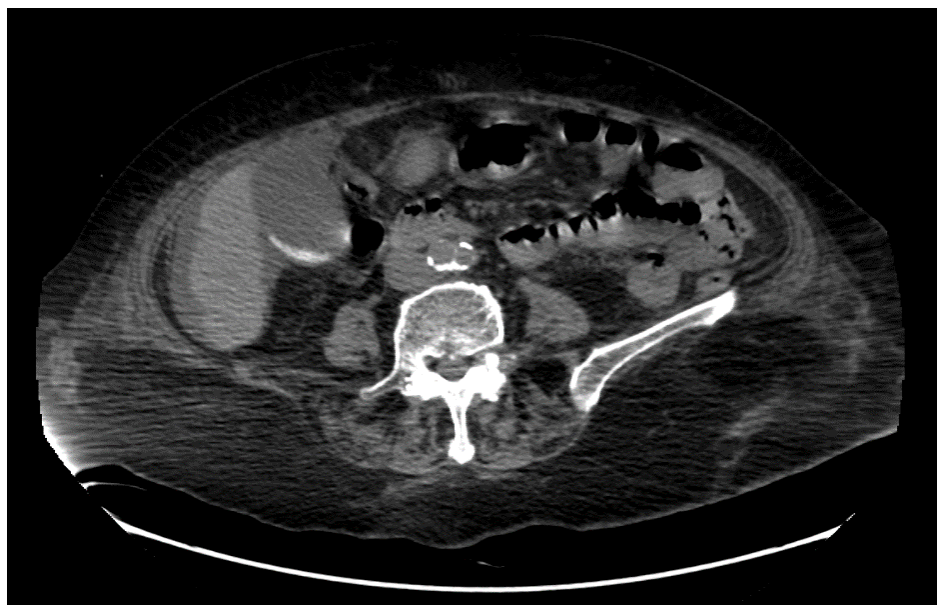
CT-scan showing a diffuse thickening of the gallbladder and inflammatory pericholecystic fluid.

This condition was conservatively treated with intravenous antibiotic therapy with renal adjusted dose of piperacillin-tazobactam and hemodialysis for two weeks. Subsequently, kidney function improved, diuresis had an increasing glomerular filtration rate and sepsis was cured. Abdominal CT-scan performed during this hospitalization had shown a diffuse thickening of the gallbladder’s wall associated with stones as well as pericholecystic fluid ( Figure 1). The CT-scan didn’t highlight pathological findings on the liver, such as enlarged regional nodes. A dilated common bile duct with the presence, in its proximal portion, of tenuously hyperdense material was described.

Endoscopic ultrasound was performed, and it confirmed the presence of both gallbladder and common duct stones, the largest was 7 millimetres, and biliary sludge with lack of dilatation of the intrahepatic biliary tract. Several stones were removed via endoscopic retrograde cholangiopancreatography, and a nasobiliary tube was left behind. Subsequent cholangiography demonstrated the regular calibre and morphology of the cystic duct, the principal biliary tract, and the intrahepatic biliary tree. However, the gallbladder appeared distended with several little stones inside.

The patient, after 6 days from the admission, finally underwent laparoscopic cholecystectomy. Intraoperative findings showed the gallbladder with thickened walls and densely fused with the liver but without other pathological findings. No intraoperative complications occurred. Histological examination of the surgical specimen highlighted the presence of metastasis from an infiltrating ductal breast carcinoma with positive hormone receptors: Estrogen Receptors (MoAb SP1) 98%, Progesterone Receptors (MoAb 1E2) 95%, Cytoprolferative Activity (MoAb MIB-1) 10%, c-erbB2 (MoAb 4B5) score: 0. The cystic lymph node showed no evidence of metastasis. The postoperative course was regular, and the patient was transferred to a rehabilitation ward five days after surgery.

After completion of the rehabilitation program, the patient was discharged, and hormone therapy (letrozole 2.5 mg once a day) was started. The patient died 15 months later due to peritoneal and bone progression of the disease.

## Review of the literature

We conducted a systematic review in which all articles describing cases of gallbladder metastasis from breast cancer were considered eligible for inclusion. Abstracts, conference papers and studies concerning animals were excluded. No restrictions were applied to publication date or languages, if there was an English version of the article available.

A systematic search for articles published up to February 2020 using PubMed, Scopus, Google Scholar and Web of Science databases was performed, and references of articles that were retrieved in the full text were also searched. The search strategy utilized in all databases included the combination of the keywords: “gallbladder metastasis”, “breast cancer”, “acute cholecystitis”, “biliary colic”, “cholelithiasis”. A minimum number of two search keywords were utilized, one of which was always “breast cancer”.

**Figure 2.**
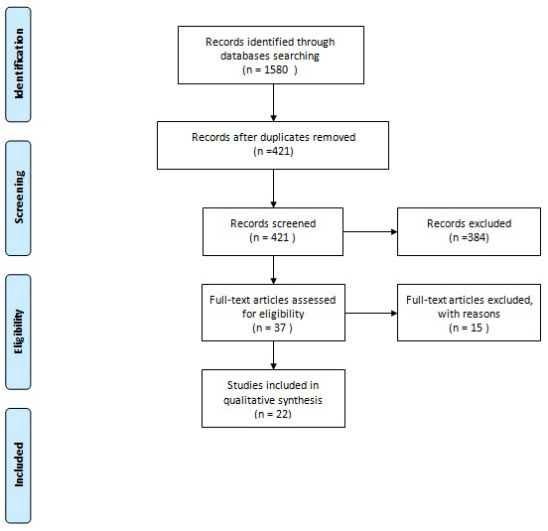
Flow diagram of articles included in the literature review.

A total of 848 potentially relevant articles were retrieved in Google Scholar, 427 in Scopus, 182 in Web Of Science and 123 in PubMed. Among these 22 studies were identified to be strictly matched with our research ( Figure 2). Our case was also included in the review.

## Discussion

In consequence of advances in medical chemotherapy and endocrine therapy in the last years, the outcomes for breast cancer are improved. Disease recurrence is more common within five years of surgery while late recurrences after more than 10 years are very uncommon. The literature outlines risk factors for late recurrence as lymph node metastases, ER + status and HER-2 negative status ^6, 7^. Breast cancer metastases occur through contiguous, lymphatic and hematogenous spread. It usually metastasizes to bone, lung, lymph nodes, liver and brain. Less frequently invaded are the endocrine organs, pericardium, abdominal cavity and eyes. Metastasis in the extrahepatic digestive system are infrequent and characteristically appear after a long latent period, which takes from three to up to 20 years ^5^.

Concerning gallbladder metastases by breast cancer, autopsy findings have shown that secondary hematogenous metastases (also from other primary organs) to the gallbladder initially generate small flat nodules below the mucosal layer. They grow as a pedunculated tumor, rarely reaching higher than several millimetres in size. The growth pattern clarifies why gallbladder metastases rarely result in clinical symptoms and that they are not diagnosed during patients’ lives. Metastatic gallbladder tumors rarely show signs; acute cholecystitis is the most frequent clinical presentation ^8^ *.* Obstructive jaundice, haemobilia, even bile peritonitis due to perforation, are seldom described. When a gallbladder metastasis is identified after surgery, the primary tumor can be not easily defined. Distinguishing between primitive gallbladder carcinoma and metastases from breast cancer is crucial for proper post-surgery therapy; in this way, immunohistochemical evaluation is necessary. The most reliable markers are gross cystic disease fluid protein such as 15 (GCDEP -15), plus cytokeratin 7, cytokeratin 20, and estrogen and progesterone receptors. Usually, their positivity is present in metastatic breast cancer, but not in all cases ^9^.

At microscopic pathological examination, metastases are often represented by small clusters and chains of neoplastic cells, commonly of the signet-ring histotype. Pathological diagnosis of metastases from lobular breast cancer can be difficult because signet-ring cells could be present in tumors originating from different organs, such as the stomach ^10^.

Our review of the literature conducted on secondary lesions of the gallbladder from breast cancer has confirmed the rarity of this disease (see Table 1 for a summary of the cases). Gallbladder metastasis is only described in 23 patients, including our case: 11 from infiltrating lobular, 7 ductal origins, 3 mixed ductal and lobular infiltration, and 3 not specified. This analysis reveals how, in most cases (12), the diagnosis of metastatic lesions was made after surgery was performed for acute cholecystitis. There was evidence of gallstones in 8 cases; 9 cases were patients who often suffer from abdominal pain and/or vomiting (symptoms of biliary colic), and so they underwent an elective cholecystectomy. Only in 2 cases, the main symptom was obstructive jaundice or bile peritonitis for necrotic gallbladder.

**Table 1.** Brief analysis of all cases of metastasis to the gallbladder we have found in the literature.

Author (year)	Age of patients (years)	Symptoms and signs	Timing of biliary symptoms after breast surgery	Gallstones	Type of breast cancer	Histology	Immunophenotype	Recurrence (months)	Exitus
Di Vita 2011 ^11^	48	Abdominal pain in the last 3 months.	3 weeks after surgery diagnosis of chronic cholecystitis at the ultrasound	No	Mixed ductal- lobular k (G3, pT2 N3 M0)	Isolated neoplastic epithelial cells in the muscular layer of the gallbladder	CK 7+, EMA +, ER+, PR+	12 SNC mets	Died 14 months after surgery
Beaver 1986 ^12^	73	Abdominal pain and vomiting (cholecystitis), also 10 months before	3 years after surgery	Yes	Not specified	Small cell tumour growing in an indian file pattern	N/A	N/A	N/A
Shah 2000 ^13^	78	Bile peritonitis for necrotic gallbladder	11 years after	Yes	Not specified	Focus of poorly differentiated adenocarcinoma characterized by gland formation and cells with eccentric cytoplasm	N/A	N/A	Died 5 days after surgery
Rubin 1989 ^14^	55	Biliary colic for 12 months	Synchronous	Yes	Lobular carcinoma	Carcinoma cells infiltrating singly an in file, mostly in the fibrous tissue deep to the muscular layer focally extended up to the mucosa	N/A	N/A	N/A
Manouras 2008 ^9^	46	Cholecystitis	2 years after surgery	Yes	Ductal	Glandular poorly differentiated metastases invading the muscular and serosa layers; scattered signet-ring cells infiltrating the mucosa	Lactalbumin +; CKT 7+; CKT 20 -; ER -; PR -	N/A	Died 1 year after surgery
Hashimoto 2016 ^15^	59	Abdominal pain (Cholecystitis)	12 years after surgery	No	Ductal (pT1c, pN0)	Poorly differentiated carcinoma full- thickness in the cystic duct and gallbladder neck	ER+; PR+; CKT 7+; her 2 -; CKT 20-; GCDEP 15 -	N/A	Died 5 years after surgery
Coletta 2014 ^16^	56	Obstructive jaundice	13 years after surgery	No	Ductal	Solid honeycombs of malignant epithelial cells localized only in the external side of the biliary duct wall; mucosa free	ER+; PR+; CK 7+; her 2 -; CK 20 -	N/A	Alive 1 year after surgery
Nair 2012 ^17^	54	Symptomatic gallstones	5 years after surgery	Yes	Lobular (T3 pN1, pMx)	The wall infiltrated by very small regular cells arranged in Indian file	N/A	N/A	Died 2 years after surgery
Al-Rawi 2012 ^18^	61	Cholecystitis	Synchronous	Yes	Lobular	Serosa and adjacent fat showed focal infiltrates of cells with rounded nuclei and small cytoplasmic vacuoles. The cells	Cytokeratins +; Epithelial Membrane antigen +; CK 7 +; ER +; CK 20 -	N/A	Died 5 years after surgery
Ebrahim 2015 ^19^	65	Asymptomatic cholelithiasis at the diagnosis of the tumour; after 2 months of chemo cholecystitis	After 2 months of therapy	Yes	Inflammatory ductal breast cancer	6–7 mm module with a pale yellow-white solid cut surface in the gallbladder wall	ER + PgR +	N/A	N/A
Molina-Barea 2014 ^20^	62	Biliary colic	After 5 years from surgery	Yes	Lobular	Infiltrated	CK 7 +; ER +	N/A	Died 12 months after surgery
Muszynska 2019 ^2^	71	Biliary colic	Few months before the diagnoses of k	Not specified	Bilateral ductal and lobular	N/A	N/A	N/A	N/A
Murguia 2006 ^5^	62	Symptomatic cholelithiasis	10 years after surgery	Yes	Ductal	Focal broad- based lesion on the mesenteric face of the body with poorly differentiated adenocarcinoma infiltration, without mucosa involvement	CK 7 +; CK 20 –; ER +0; PgR +	N/A	Died 2 years after surgery for myocardial infarction (2 months before she had done PET and CA 15.3, normal)
Mouchli 2019 ^21^	52	Acute cholecystitis	1 year after surgery	No	Ductal	N/A	N/A	N/A	Died several days after the surgery
Riaz 2012 ^22^	42	Asymptomatic (finding of a focal area of thickening in gallbladder’s body during the US for staging)	Synchronous	No	Lobular	Cords and nests of malignant cells showing moderate amount of eosinophilic cytoplasm containing irregular hyperchromatic nuclei; indian file pattern is present	Cytoplasmic mucin +; CK 7 +; CK 20 –; E-cadherin –; ER +; PgR+	N/A	Stable disease until her last follow up
Markelov 2011 ^23^	67	Nausea + weight loss (gallbladder dyskinesia)	6 years after surgery	Not specified	Lobular with some foci of in situ ductal	Foci of tumour with a single file arrangement present outside the muscularis propria and some tumour cells within the muscolaris propria	ER +; PgR +; Ki67 +; HER 2 -	N/A	N/A
Zagouri 2007 ^24^	59	Acute cholecystitis	20th month after surgery	Yes	Bilateral synchronous lobular + ductal	The muscular layer and adventitia of the body of gallbladder was infiltrated	ER +; PgR –; CK AE1/AE3 +	N/A	Alive 1 year after surgery
Abdelilah 2014 ^25^	45	Acute cholecystitis	3 months after surgery	Yes	Lobular (T3 N1 M0)	1.5 cm palpable mass	ER + PgR+	N/A	N/A
Zamkowski 2017 ^26^	64	Acute cholecystitis	Synchronous	No	Lobular bilateral	Not described	ER +; PgR – ; HER2 –; Ki67 +	N/A	Alive at the moment of the drafting of the article
Fleres 2014 ^27^	83	Biliary colic with gallstones also in VBP	Synchronous	Yes	Lobular	Parietal infiltration	Ck AE1/AE3 +; CK 7 +; CK 8 +; ER +; PgR -	N/A	Alive 3 years after surgery
Herrera 2010 ^28^	46	Acute cholecystitis	10 years after surgery	Yes	Lobular	Not specified	N/A	N/A	N/A
Machida 2007 ^29^	53	Acute cholecystitis	18 years after surgery	No	Lobular	Necrotic change was seen until the muscular layer; white nodules were detected in the submucosal layer of the neck	N/A	N/A	N/A
Our experience	86	Acute cholecystitis	21 years after surgery	Yes	Ductal	Parietal infiltration	ER +; PgR +; Mib 1 10%; HER2 0	13 months peritoneal and bone	Died 15 months after surgery

Instrumental diagnostics are useless as they do not show significant data on gallbladder walls that are suspicious for malignancy; the identification of the neoplastic disease is possible only after surgery during histological examination of the specimen, as was shown in our case. From the analysis of the cases described in the literature, it follows that the most frequent tumor histology associated with gallbladder metastasis by breast cancer is infiltrating lobular carcinoma.

This review shows how the detection of gallbladder metastasis usually occurs any time after the surgery for the primary tumor. In essence, we would highlight that in 6 cases, it happened after more than 10 years from primary surgery, in 7 cases between 1 and 6 years, and 3 cases within the first year. Only in 6 cases was the detection of breast cancer and gallbladder metastasis synchronous.

## Conclusions

This report emphasizes the importance of long-term follow up in patients with a history of breast cancer.

Our experience and data from the literature suggest carefully evaluating every anomaly observed during routine staging examinations, even when apparently due to benign, mild disease. Metastatic disease always should be included in the differential diagnosis of a patient with a history of invasive breast cancer and new onset of abdominal pain. Conventional methods of documenting gallbladder disease are nonspecific concerning the malignant disease. This may pose a diagnostic challenge in patients with abdominal symptoms after resection of malignancies, also because they need to be aggressively treated as it can improve the poor prognosis of these cases. From our case and literature review, we recommend the following:

1. Consider the oncological story of the patients in the emergency setting;
2. Metastatic disease should be included in the differential diagnosis in patients with a history of breast cancer.

## Consent

Written informed consent for publication of clinical details and clinical images was obtained from the patient on admission to hospital prior to the patient’s death.

## Data availability

No data is associated with this article.

## References

[1] Yoon WJ Yoon YB Kim YJ: Metastasis to the gallbladder: a single-center experience of 20 cases in South Korea. *World J Gastroenterol.* 2009;15(38):4806–4809. 10.3748/wjg.15.4806 19824115 PMC2761559

[2] Muszynska C Lundgren L Andersson R: Incidental metastases and lymphoma of the gallbladder - an analysis of ten rare cases identified from a large national database. *Scand J Gastroenterol.* 2019;54(3):350–358. 10.1080/00365521.2019.1588363 31035806

[3] Terasaki S Nakanuma Y Terada D: Metastasis of hepatocellular carcinoma to the gallbladder presenting massive intraluminal growth: Report of an autopsy case. *J Clin Gastroenterol.* 1990;12(6):714–5. 10.1097/00004836-199012000-00028 2176231

[4] Chan KW: Review of 253 cases of significant pathology in 7,910 cholecystectomies in Hong Kong. *Pathology.* 1988;20(1):20–3. 10.3109/00313028809085191 3374970

[5] Murguia E Quiroga D Canteros G: Gallbladder metastases from ductal papillary carcinoma of the breast. *J Hepatobiliary Pancreat Surg.* 2006;13(6):591–3. 10.1007/s00534-006-1119-z 17139439

[6] Nishimura R Osako T Nishiyama Y: Evaluation of factors related to late recurrence—later than 10 years after the initial treatment—in primary breast cancer. *Oncology.* 2013;85(2):100–10. 10.1159/000353099 23867253

[7] Ven Ustaalioglu BB Balvan O Bilici A: The differences of clinicopathological factors for breast cancer in respect to time of recurrence and effect on recurrence-free survival. *Clin Transl Oncol.* 2015;17(11):895–902. 10.1007/s12094-015-1323-x 26081286

[8] Bush E Geradts J O'Connor T: An unusual cause of cholecystitis? *Am J Clin Oncol.* 2005;28(5):529–30. 10.1097/01.coc.0000162421.40734.8a 16199997

[9] Manouras A Lagoudianakis EE Genetzakis M: Metastatic breast carcinoma initially presenting as acute cholecystitis: a case report and review of the literature. *Eur J Gynaecol Oncol.* 2008;29(2):179–81. 18459559

[10] Merino MJ Livolsi VA: Signet ring carcinoma of the female breast: A clinicopathologic analysis of 24 cases. *Cancer.* 1981;48(8):1830–7. 10.1002/1097-0142(19811015)48:8<1830::aid-cncr2820480821>3.0.co;2-h 6269726

[11] Di Vita M Zanghì A Lanzafame S: Gallbladder metastases of breast cancer: from clinical-pathological patterns to diagnostic and therapeutic strategy. *Clin Ter.* 2011;162(5):451–6. 22041804

[12] Beaver BL Denning DA Minton JP: Metastatic breast carcinoma of the gallbladder. *J Surg Oncol.* 1986;31(4):240–2. 10.1002/jso.2930310404 3724179

[13] Shah RJ Koehler A Long JD: Bile peritonitis secondary to breast cancer metastatic to the gallbladder. *Am J Gastroenterol.* 2000;95(5):1379–81. 10811372 10.1111/j.1572-0241.2000.02054.x

[14] Rubin A Tate JJ: Breast carcinoma metastatic to the gallbladder. *J Clin Pathol.* 1989;42(11):1223–4. 10.1136/jcp.42.11.1223 2584434 PMC501987

[15] Hashimoto M Koide K Arita M: Acute acalculous cholecystitis due to breast cancer metastasis to the cystic duct. *Surg Case Rep.* 2016;2(1): 111. 10.1186/s40792-016-0239-1 27730536 PMC5059227

[16] Coletta M Montalti R Pistelli M: Metastatic breast cancer mimicking a hilar cholangiocarcinoma: case report and review of the literature. *World J Surg Oncol.* 2014;12:384. 10.1186/1477-7819-12-384 25515643 PMC4301035

[17] Nair MS Phillips BL Jain G: Gall Bladder Metastasis from Breast Cancer Masquerading Symptomatic Cholelithiasis. *J Gastrointest Cancer.* 2012;43 Suppl 1:S215–6. 10.1007/s12029-012-9382-5 22418771

[18] Al-Rawi H Al-Jafari M Mathew H: Metastatic breast cancer mimicking cholecystitis. A rare clinical presentation. *Saudi Med J.* 2012;33(10):1128–30. 23047208

[19] Ebrahim H Graham D Rice D: Inflammatory metastatic breast cancer with gallbladder metastasis: an incidental finding. *J Community Support Oncol.* 2015;13(7):256–9. 10.12788/jcso.0154 26270542

[20] Molina-Barea R Rios-Peregrina RM Slim M: Lobular breast cancer metastasis to the colon, the appendix and the gallbladder. *Breast Care (Basel).* 2014;9(6):428–30. 10.1159/000368430 25759626 PMC4317682

[21] Mouchli M Grider DJ Yeaton P: Gallbladder Metastases: A Report of Two Cases. *Case Rep Oncol.* 2019;12(1):235–240. 10.1159/000497818 31011322 PMC6465751

[22] Riaz N Ahmed R Afzal S: Breast carcinoma with asymptomatic metastasis to the gallbladder. *Singapore Med J.* 2012;53(7):e136–8. 22815028

[23] Markelov A Taheri H Vunnamadala K: Biliary dyskinesia as a rare presentation of metastatic breast carcinoma of the gallbladder: a case report. *Case Rep Pathol.* 2011;2011:806570. 10.1155/2011/806570 22937393 PMC3420450

[24] Zagouri F Sergentanis TN Koulocheri D: Bilateral synchronous breast carcinomas followed by a metastasis to the gallbladder: a case report. *World J Surg Oncol.* 2007;5:101. 10.1186/1477-7819-5-101 17848197 PMC2075501

[25] Abdelilah B Mohamed O Yamoul R: Acute cholecystitis as a rare presentation of metastatic breast carcinoma of the gallbladder: A case report and review of the literature. *Pan Afr Med J.* 2014;17:216. 10.11604/pamj.2014.17.216.3911 25237413 PMC4163176

[26] Zamkowski M Kąkol M Makarewicz W: Patient with metastatic breast cancer presenting as acute cholecystitis with one-year survival on hormonotherapy. *Pol Przegl Chir.* 2017;89(4):46–49. 10.5604/01.3001.0010.4063 28905808

[27] Fleres F Rossitto M Foti A: Metastasis of the gallbladder from the breast cancer. *Ann Ital Chir.* 2014;85: pii: S2239253X14023470. 25707544

[28] Herrera FA Jr Hassanein AH Cosman BC: Breast carcinoma metastatic to the gallbladder and urinary bladder. *Eur Rev Med Pharmacol Sci.* 2010;14(10):883–6. 21222376

[29] Machida D Yukawa N Gohda M: A case of metastatic breast carcinoma of the gallbladder. *Jpn J Gastroenterol Surg.* 2007;40:56–62. 10.5833/jjgs.40.56

