# Supplementary material for: Case Report: Metastatic breast cancer to the gallbladder
Source: F1000Res. 2020 May 11;9:343. [Version 1] doi: 10.12688/f1000research.23469.1 (PMC7610173; doi:10.12688/f1000research.23469.1)
Supplement: Peer review history

# Peer review: Reviewer response for version 1

**DOI:** 10.5256/f1000research.25900.r72530

**Published:** 2020-10-15

**Authors:** Francesco Fleres

**Affiliations:** 1 Department of Human Pathology, University of Messina, Messina, Italy; 2 ASST Valtellina e Alto Lario, Sondrio Hospital, Sondrio, Italy

- The authors have reported a very interesting and rare case. They have described very well the rarity of the case and oncologic history.
- Moreover they have performed an interesting and very complete analysis and review of the literature. It can be accepted as it is.
- Good level of language.
- It would be interesting if the authors can describe and argue better on the histologic exam.

I would like to ask some opinions from the authors:

1. Which was the dimension of the metastasis?
2. Where was it located, on peritoneal surface or on liver's bed?
3. How was the gallbladder extracted? Did you used a endobag?
4. Was there evident an infiltration of the cistic duct?
5. On their opinion and review of literature, should it be considered a more extensive intervention on liver bed as incidental gallbladder cancer?
6. The oncologic therapy was performed as hormone therapy and not chemotherapy due the patient's general condition?

This report and review of literature in very interesting and it can be accepted.

Are enough details provided of any physical examination and diagnostic tests, treatment given and outcomes?

Yes

Is the case presented with sufficient detail to be useful for other practitioners?

Yes

Is sufficient discussion included of the importance of the findings and their relevance to future understanding of disease processes, diagnosis or treatment?

Yes

Is the background of the case’s history and progression described in sufficient detail?

Yes

Reviewer Expertise:

General surgeon, Oncologic surgeon, colorectal cancer, gastric cancer, Hepato-biliary-pancreatic surgeon. Hipec, Sarcoma, Hernia, Laparoscopic surgery, robotic surgery, peritoneal carcinosis.

I confirm that I have read this submission and believe that I have an appropriate level of expertise to confirm that it is of an acceptable scientific standard.

# Peer review: Reviewer response for version 1

**DOI:** 10.5256/f1000research.25900.r64929

**Published:** 2020-07-30

**Authors:** Giorgio Ercolani

**Affiliations:** 1 Department of Medical and Surgical Sciences - DIMEC, Alma Mater Studiorum - University of Bologna, Bologna, Italy

I believe that the case-report is interesting, well reported and should be accepted.

 However, I have a minor concern:

- Did the authors perform a liver resection of the gallbladder bed? Is it reported in the literature? Since most of the reported patients died within 2 years from cholecystectomy, the author should discuss if in the finding of incidental metastases from breast cancer, a more extensive procedure (radical cholecystectomy with resection of the gallbladder bed) should be applied similarly to the incindetally gallbladder carcinoma. 

Are enough details provided of any physical examination and diagnostic tests, treatment given and outcomes?

Yes

Is the case presented with sufficient detail to be useful for other practitioners?

Partly

Is sufficient discussion included of the importance of the findings and their relevance to future understanding of disease processes, diagnosis or treatment?

Yes

Is the background of the case’s history and progression described in sufficient detail?

Yes

Reviewer Expertise:

:Liver tumor; liver surgery

I confirm that I have read this submission and believe that I have an appropriate level of expertise to confirm that it is of an acceptable scientific standard.
